# Assessing the Willingness to Work in Rural Areas and Associated Factors Among Medical Students at King Saud University in Riyadh, Saudi Arabia

**DOI:** 10.7759/cureus.101248

**Published:** 2026-01-10

**Authors:** Nada Alobaid, Turki BinMoammar, Abdulrahman A Alotaibi, Basel A Fakeeha, Ali M Almatri, Mohammed Alwahibi, Alanoud Almufarrej, Noorah A Aldubaib, Esraa Alnazzawi

**Affiliations:** 1 Department of Family and Community Medicine, College of Medicine, King Saud University, King Saud University Medical City, Riyadh, SAU; 2 College of Medicine, King Saud University, Riyadh, SAU

**Keywords:** career intention, healthcare workforce, medical students, rural health, saudi arabia, workforce distribution

## Abstract

Background: Maldistribution of the health workforce remains a major barrier to equitable healthcare access, particularly in rural areas. Medical students represent the future physician workforce, and their career intentions are critical for addressing rural healthcare gaps.

Objective: To assess the willingness of medical students and interns at King Saud University (KSU) to work in rural areas and to identify factors associated with this willingness.

Methods: A cross-sectional study was conducted among medical students and interns at KSU using a structured self-administered questionnaire. Data collected included sociodemographic characteristics, educational background, rural exposure, career motivations, and willingness to work in rural areas. Data were analyzed using descriptive statistics and logistic regression analyses to identify factors associated with willingness to work in rural settings.

Results: A total of 372 students participated. Only 42 (11.3%) expressed a positive intention to work in rural areas, while 302 (81.2%) preferred urban practice. The most significant deterrents to rural practice were poor clinical infrastructure (44, 14.3%), lack of training opportunities (41, 13.4%), limited career progression (32, 10.4%), and insufficient financial incentives (27, 8.7%). The most influential incentives included a base salary increase of 30% or more (223, 59.9%), a supportive workplace and management (172, 46.2%), and improved infrastructure and medical supplies (171, 46.0%). Multivariable logistic regression showed that senior academic year was negatively associated with willingness, while older age (25-34 years) and prior rural residential experience were positive predictors.

Conclusion: Medical students at KSU demonstrate very low willingness to work in rural areas. Addressing professional, infrastructural, and financial barriers, along with early rural exposure and decentralized training opportunities, may help improve rural workforce distribution in Saudi Arabia.

## Introduction

Achieving universal health coverage remains a central objective of the global health agenda; however, this goal continues to be substantially hindered by the persistent maldistribution of the health workforce [1,2]. This challenge is particularly evident in the disproportionate concentration of healthcare professionals in urban areas, leaving rural and remote communities underserved and facing limited access to essential health services [3]. The World Health Organization (WHO) has identified health workforce maldistribution as a major barrier to health equity, noting that while nearly half of the global population resides in rural areas, only a small proportion of healthcare professionals choose to practice in these settings [4,5]. This imbalance contributes to poorer health outcomes in rural populations, including higher rates of preventable morbidity and mortality, underscoring the urgent need for effective strategies to recruit and retain healthcare workers in underserved areas [6].

In the Kingdom of Saudi Arabia (KSA), the healthcare system is undergoing a significant transformation aligned with Saudi Vision 2030, which emphasizes improving the quality, efficiency, and equitable distribution of healthcare services across the country [7]. Despite substantial investments in healthcare infrastructure and medical education, the unequal distribution of the medical workforce remains a critical concern [8]. Major urban centers, such as Riyadh, are well-equipped with specialized physicians and advanced healthcare facilities, whereas peripheral and rural regions continue to experience shortages in healthcare services, basic medical resources, and qualified physicians [9]. This geographic imbalance risks widening existing health disparities and undermining Vision 2030 objectives [10].

Medical students and interns represent a critical pipeline for the future physician workforce [11]. Decisions regarding rural practice are influenced by personal, professional, and educational factors, including quality of life, financial incentives, and exposure to rural training [12-14]. Positive rural clinical experiences have been shown to significantly increase the likelihood of rural practice [15]. King Saud University (KSU), located in Riyadh, provides students with extensive urban-based resources, which may influence perceptions of rural practice. This study aims to assess the willingness of KSU medical students and interns to work in rural areas and identify associated factors influencing these intentions.

## Materials and methods

Study design

A cross-sectional survey-based study was conducted among medical students and interns at King Saud University, Riyadh, Saudi Arabia.

Study population

The study population included medical students and interns enrolled at King Saud University during the study period. Internship represents a mandatory 12-month supervised clinical training year undertaken immediately after completion of undergraduate medical education, and is required for completion of the MBBS (Bachelor of Medicine, Bachelor of Surgery) degree prior to independent medical practice.

Sample size

The sample size was calculated using the single population proportion formula, with a 95% confidence level and a margin of error of 5%. In the absence of prior local estimates regarding willingness to work in rural areas, a proportion of 50% was assumed to obtain the maximum required sample size. Based on a total population of 1,509 medical students and interns at King Saud University, the minimum required sample size was calculated to be 307 participants. A total of 372 participants completed the survey and were included in the final analysis.

Study measures

Data were collected using a structured, self-administered questionnaire distributed electronically that assessed sociodemographic characteristics, educational level, prior rural exposure, willingness to work in rural areas, perceived deterrents, and incentives influencing career decisions. The questionnaire was adopted from a previously published and peer-reviewed study by Lori et al. (2012) and was used without major modifications [16].

Ethics statement

The study was approved by the Institutional Review Board of King Saud University (IRB approval number: E-24-9443). Electronic informed consent was obtained from all participants prior to participation.

Statistical analysis

Data were analyzed using IBM SPSS Statistics version 31 (IBM Corp., Armonk, NY). Categorical variables were summarized as frequencies and percentages, while numerical variables were presented as medians with interquartile ranges (IQR) and means with standard deviations (SD). Relative scores were calculated using a weighted ranking approach. Participants ranked their top three factors, with first, second, and third-ranked choices assigned weights of 3, 2, and 1, respectively. The sum of weighted percentages was divided by six to generate the relative score.

Logistic regression analysis was performed to identify factors associated with willingness to work in rural areas. The outcome variable was derived from the survey question assessing future work intentions in deprived (rural) areas, which included four response options: “I will definitely not work in a deprived area,” “I am unlikely to work in a deprived area,” “I am likely to work in a deprived area,” and “I will definitely work in a deprived area.” For regression analysis, responses were dichotomized as willing (likely/definitely; coded as 1) and not willing (unlikely/definitely not; coded as 0). Both univariate and multivariable models were used.

All variables considered a priori to be clinically and contextually relevant based on existing literature (including age, gender, academic year, history of rural residence, and type of secondary school) were entered into the multivariable model. Model assumptions were assessed prior to analysis. Independence of observations was assumed as each participant completed the questionnaire once. Multicollinearity was evaluated using variance inflation factors (VIFs), all of which ranged between 1.03 and 1.31, indicating no significant multicollinearity. Model fit was assessed using the Hosmer-Lemeshow goodness-of-fit test (p = 0.930). Potential confounders were retained in the multivariable model, and adjusted odds ratios with 95% confidence intervals are reported. A p-value of <0.05 was considered statistically significant.

## Results

A total of 372 medical students participated in the study. The sociodemographic characteristics of the participants are presented in Table 1. Only 40 (10.8%) reported having lived in a rural area since childhood.

**Table 1:** Sociodemographic characteristics of the studied medical students (n = 372).

	n	%
Current year in the university		
1^st^ year	42	11.3%
2^nd^ year	44	11.8%
3^rd^ year	60	16.1%
4^th^ year	44	11.8%
5^th^ year	106	28.5%
Internship	76	20.4%
Gender		
Male	204	54.8%
Female	168	45.2%
Age		
18-24	339	91.1%
25-34	33	8.9%
Residence		
Urban area (settlement with a population of more than 5000)	362	97.3%
Peri-urban area (adjacent to an urban area)	7	1.9%
Rural area (settlement with a population of less than 5000)	3	0.8%
Birth region		
Central Region	292	78.5%
Southern Region	24	6.5%
Western Region	23	6.2%
Eastern Region	21	5.6%
Northern Region	7	1.9%
International	5	1.3%
Birth area		
Urban area (settlement with a population of more than 5000)	325	87.4%
Peri-urban area (adjacent to an urban area)	24	6.5%
Rural area (settlement with a population of less than 5000)	15	4.0%
Don’t know	8	2.2%
Secondary school (n = 369)		
Private	190	51.5%
Public	178	48.2%
Don't know	1	0.3%
Secondary school region (n = 370)		
Central Region	330	89.2%
Eastern Region	21	5.7%
Southern Region	8	2.2%
Western Region	7	1.9%
Northern Region	3	0.8%
International	1	0.3%
Secondary school area		
Urban area (settlement with a population of more than 5000)	345	92.7%
Peri-urban area (adjacent to an urban area)	19	5.1%
Rural area (settlement with a population of less than 5000)	5	1.3%
Don’t know	3	0.8%
From the age of 5 onwards, have you lived in a rural area? (n = 366)		
No	319	87.2%
Yes	40	10.9%
Don't know	7	1.9%
Total time of living in rural area (years) (n= 39)	Median (IQR)	7 (14)
Rural area region (n = 39)		
Central Region	19	5.1%
Southern region	13	3.5%
Eastern Region	3	0.8%
Western Region	3	0.8%
Northern Region	1	0.3%
International	2	0.5%
None of the above	2	0.5%
Currently married (n = 365)		
No	351	96.2%
Yes	14	3.8%
Number of children (n = 12)	Median (IQR)	0 (0.8)

Only 42 (11.3%) of the participants expressed willingness to work in rural areas, while 302 (81.2%) preferred urban practice. Key deterrents included poor clinical infrastructure, lack of training opportunities, limited career advancement, and inadequate financial incentives. Salary and infrastructure ranked as the most influential career factors.

Multivariable logistic regression analysis showed that senior students and interns were significantly less willing to work in rural areas. Older age (25-34 years) and previous rural residence were independently associated with increased willingness.

Table 1 presents the sociodemographic characteristics of the participating medical students who are currently enrolled in King Saud University. Due to optional responses for some variables, denominators varied across certain analyses.

The largest proportions were 5th-year students (106, 28.5%), followed by interns (76, 20.4%), indicating that the sample is slightly weighted toward senior students. The gender distribution revealed a moderate male predominance: 204 (54.8%) males vs. 168 (45.2%) females. The vast majority of participants were young adults, with 339 (91.1%) aged 18-24 years, which aligns with the typical age range for undergraduate medical education.

Most participants reported currently living in urban areas (362, 97.3%), with similarly high proportions for birth area (325, 87.4%) and secondary school area (345, 92.7%). Only a very small percentage had lived in rural areas, with 40 (10.8%) reporting any rural residence since age five, and the recorded duration of rural residence showed considerable variability (median = 7 years).

With respect to geographical distribution, the majority of students originated from the Central Region, both in terms of birth region (292, 78.5%) and secondary school region (330, 88.7%), reflecting the university’s primary catchment area.

Regarding marital status, most participants were not married (351, 94.4%), consistent with their young age, and only a very small fraction had children.

Overall, the table describes a young, predominantly urban, Central Region-based medical student population with limited rural exposure.

Table 2 summarizes the original and current motivations for studying medicine among the participating medical students based on their first, second, and third-ranked choices, along with relative scores.

**Table 2:** Original and current motivation for studying medicine among the studied medical students (n = 372).

Original motivation for studying medicine	1^st^	2^nd^	3^rd^	Relative score
Desire to help others	116 (31.2%)	62 (16.7%)	33 (8.9%)	22.7%
Job security and lifestyle	68 (18.3%)	69 (18.5%)	54 (14.5%)	17.7%
Interest in health as a subject matter	58 (15.6%)	32 (8.6%)	42 (11.3%)	12.6%
Income of doctors	23 (6.2%)	55 (14.8%)	54 (14.5%)	10.5%
Social status/prestige of doctors	14 (3.8%)	38 (10.2%)	53 (14.2%)	7.7%
Parents proposed it to me	18 (4.8%)	18 (4.8%)	28 (7.5%)	5.3%
Inspired by a role model	18 (4.8%)	24 (6.5%)	15 (4%)	5.2%
Give back to your home community or country	14 (3.8%)	23 (6.2%)	28 (7.5%)	5.2%
Research opportunities	3 (0.8%)	13 (3.5%)	7 (1.9%)	1.9%
Opportunity to travel and work internationally	3 (0.8%)	10 (2.7%)	11 (3%)	1.8%
Loss of a loved one	4 (1.1%)	3 (0.8%)	9 (2.4%)	1.2%
Use new, cutting-edge technologies	5 (1.3%)	2 (0.5%)	6 (1.6%)	1.1%
Other	9 (2.4%)	2 (0.5%)	9 (2.4%)	1.8%
Current motivations for studying medicine	1^st^	2^nd^	3^rd^	Relative score
Desire to help others	124 (33.3%)	64 (17.2%)	34 (9.1%)	23.9%
Job security and lifestyle	69 (18.5%)	52 (14%)	56 (15.1%)	16.4%
Interest in health as a subject matter	36 (9.7%)	47 (12.6%)	46 (12.4%)	11.1%
Income of doctors	32 (8.6%)	53 (14.2%)	59 (15.9%)	11.7%
Social status/prestige of doctors	10 (2.7%)	32 (8.6%)	43 (11.6%)	6.2%
Give back to your home community or country	22 (5.9%)	23 (6.2%)	21 (5.6%)	6.0%
Inspired by a role model	7 (1.9%)	19 (5.1%)	22 (5.9%)	3.6%
Research opportunities	9 (2.4%)	12 (3.2%)	10 (2.7%)	2.7%
Use new, cutting-edge technologies	6 (1.6%)	12 (3.2%)	7 (1.9%)	2.2%
Opportunity to travel and work internationally	2 (0.5%)	15 (4%)	10 (2.7%)	2.0%
Loss of a loved one	8 (2.2%)	2 (0.5%)	12 (3.2%)	1.8%
Other	11 (3%)	5 (1.3%)	16 (4.3%)	2.7%

Regarding the original motivation for studying medicine, the most prominent motivating factor was the desire to help others, which consistently appeared as the top-ranked reason (110 of 353, 31.2% as first choice) and had the highest overall relative score (22.7). Job security and lifestyle emerged as the second most influential motivator, with a relative score of 17.7, followed by interest in health as a subject matter (relative score = 12.6).

Financial motivations were less dominant, with the income of doctors having a relative score of 10.5. Motivations related to social status, parental influence, role models, and giving back to one’s home community showed moderate importance, with relative scores of 7.7, 5.3, 5.2, and 5.2, respectively.

Less commonly cited motivations included research opportunities (relative score = 1.9), opportunities to travel internationally (relative score = 1.8), loss of a loved one (relative score = 1.2), use of cutting-edge technologies (relative score = 1.1), and other (relative score = 1.8), each contributing relatively small proportions to the overall motivation structure.

Regarding the current motivation for studying medicine, the most prominent current motivation remained the desire to help others, with 112 of 336 students (33.3%) ranking it first and a relative score of 23.9, slightly higher than in the original motivation.

Job security and lifestyle continued to be the second most important motivator, with a relative score of 16.4, followed by interest in health as a subject matter (relative score = 11.1). Financial considerations, represented by the income of doctors, showed an increase in importance relative to the original motivation, with a relative score of 11.7.

Moderately cited motivations included giving back to one’s home community or country (relative score = 6.0), social status/prestige of doctors (relative score = 6.2), and inspiration by a role model (relative score = 3.6).

Less commonly cited current motivations were research opportunities (relative score = 2.7), use of cutting-edge technologies (relative score = 2.2), opportunity to travel and work internationally (relative score = 2.0), loss of a loved one (relative score = 1.8), and other (relative score = 2.7), all contributing relatively small proportions to the overall motivation structure.

As shown in Table 3, the majority of students (319, 85.8%) reported that they were not under any obligation or had not made any commitment to return to a specific area to work after graduation. Only a small proportion (25, 6.7%) indicated that they did have such obligations.

**Table 3:** Work obligation characteristics of the studied medical students (n = 372). Categorical data are presented as frequency (%).

	n	%
Are you under any obligation or have you made any commitment to return to a specific area to work after graduation? (n = 344)		
No	319	85.8%
Yes	25	6.7%
Work obligation duration (n = 14)		
1-10 years	6	1.6%
>10-20 years	2	0.5%
Minimum 5 years, but may extend for life	1	0.3%
The rest of life	4	1.1%
Work obligation region (n = 25)		
Central Region	16	4.3%
Western Region	2	0.5%
Southern Region	2	0.5%
Eastern Region	1	0.3%
Northern Region	1	0.3%
International	3	0.8%
Work obligation region area (n = 25)		
Urban area (settlement with a population of more than 5000)	23	6.2%
Peri-urban area (adjacent to an urban area)	2	0.5%

Among students with work obligations (n = 25), the duration of commitment varied. Most reported obligations ranging from 1-10 years (6, 24.0%), while fewer reported obligations of >10-20 years (2, 8.0%), followed by a minimum of five years but potentially extending for life (1, 4.0%) and for the rest of life (4, 16.0%).

Regarding the region of work obligation among those with obligations (n = 25), most were assigned to the Central Region (16, 64.0%), with smaller proportions in the Western and Southern Regions (two (8.0%) each), Eastern and Northern Regions (one (4.0%) each), and international locations (3, 12.0%). Most obligations were in urban areas (23, 92.0%), with a minority in peri-urban areas (2, 8.0%), and none were reported in rural areas.

As shown in Table 4, the majority of students (302, 81.2%) expressed a preference to work in urban areas, with smaller proportions preferring peri-urban areas (22, 5.9%) or rural areas (3, 0.8%). A minority of students (13, 3.5%) reported being uncertain about their preferred work location.

**Table 4:** Work location preferences after graduation among the studied medical students (n = 372). Categorical data are presented as frequency (%).

	n	%
Where do you hope to work after graduation? (n = 340)		
Urban area (settlement with a population of more than 5000)	302	81.2%
Peri-urban area (adjacent to an urban area)	22	5.9%
Rural area (settlement with a population of less than 5000)	3	0.8%
Don't know	13	3.5%
After graduation, hoped work region (n = 339)		
Central Region	295	79.3%
Eastern Region	16	4.3%
Western Region	9	2.4%
Northern Region	5	1.3%
Southern Region	5	1.3%
International	9	2.4%

Regarding the preferred region of work (n = 339), most students hoped to work in the Central Region (295, 79.3%), followed by the Eastern Region (16, 4.3%), Western Region (9, 2.4%), Northern Region (5, 1.3%), Southern Region (5, 1.3%), and international locations (9, 2.4%).

As shown in Table 5, the majority of students (312, 83.9%) reported no participation in outreach or service activities in deprived areas. A small proportion (15, 4.0%) indicated that they had participated, while nine (2.4%) preferred not to disclose their experience.

**Table 5:** Outreach and service experience in deprived areas during medical training among the studied medical students (n = 372).

	n	%
During your medicine studies thus far, have you done outreach or service in a deprived area? (n = 336)		
No	312	83.9%
Yes	15	4.0%
Rather not to say	9	2.4%
Duration of time spent doing outreach or service in a deprived area during medical training (n = 12)		
0-5 weeks	9	2.4%
>5 weeks	3	0.8%

Among those who participated (n = 12), most spent 0-5 weeks (9, 75.0%), while three (25.0%) spent >5 weeks, which corresponds to nine (2.4%) and three (0.8%) of the total sample.

Table 6 summarizes the factors considered most important by medical students when selecting their future work location, based on their first, second, and third-ranked choices and relative scores.

**Table 6:** The most important factors in selecting future work location among the studied medical students (n = 372).

Factors most important in selecting a future work location	1^st^ (n = 319)	2^nd^ (n = 319)	3^rd^ (n = 318)	Relative score
Income potential	19.6%	8.1%	8.3%	13.9%
Professional support and mentorship	15.3%	13.4%	5.4%	13.0%
Access to training opportunities	9.4%	8.9%	10.8%	9.5%
Proximity to the city	9.4%	7.0%	7.8%	8.3%
Quality of clinic facilities	5.1%	10.2%	6.7%	7.1%
Availability of material resources (supplies, equipment)	6.5%	7.0%	5.9%	6.6%
Exposure to a challenging work environment (clinical skills)	4.3%	5.1%	5.6%	4.8%
Access to technology	2.2%	8.9%	4.0%	4.7%
Ability to return to school to obtain an advanced specialty in a specialized area (e.g., ENT and ophthalmology)	2.7%	4.3%	5.4%	3.7%
Quality of housing	1.9%	4.0%	7.0%	3.5%
Quality of education for children	1.6%	1.6%	7.0%	2.5%
Promotion prospects	0.8%	4.8%	3.2%	2.5%
Ability to return to the university to pursue a degree program	2.4%	2.2%	2.2%	2.3%
Don't know/rather not say	1.3%	3.0%	3.0%	2.2%
Employment for spouse/partner	0.5%	1.6%	2.2%	1.2%
Other	2.7%	0.3%	1.1%	1.6%

Income potential ranked highest, with the greatest relative score (13.9%), followed by professional support and mentorship (13.0%). Access to training opportunities (9.5%) and proximity to the city (8.3%) were also highly ranked factors.

Moderately ranked factors included quality of clinic facilities (7.1%), availability of material resources such as supplies and equipment (6.6%), exposure to a challenging work environment (4.8%), and access to technology (4.7%).

Lower-ranked factors included the ability to return to school for advanced specialty training (3.7%), quality of housing (3.5%), quality of education for children (2.5%), promotion prospects (2.5%), ability to return to university for further studies (2.3%), uncertainty regarding preferences (2.2%), employment opportunities for spouse or partner (1.2%), and other factors (1.6%).

**Figure 1:**
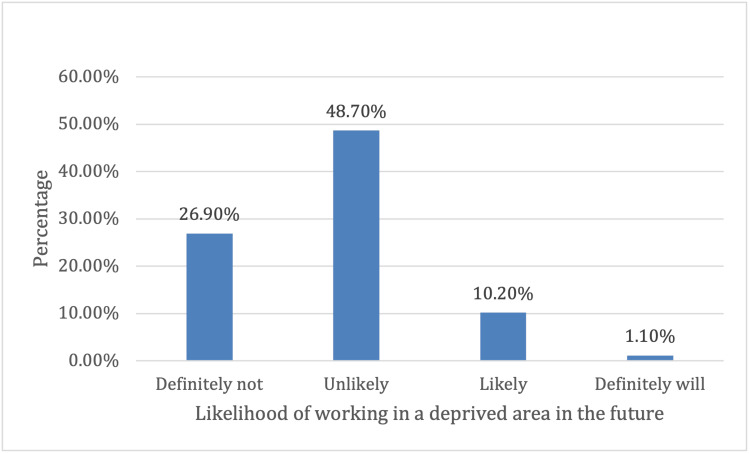
Likelihood of working in a deprived area in the future.

As shown in Table 7, the majority of students expressed a low likelihood of working in a deprived area. Specifically, 100 (26.9%) stated that they would definitely not work in a deprived area, while 181 (48.7%) reported that they were unlikely to do so. Only a small proportion of students reported a positive inclination, with 38 (10.2%) indicating they were likely and four (1.1%) stating they would definitely work in a deprived area. The distribution of students’ likelihood of working in rural areas is illustrated in Figure 1.

**Table 7:** Likelihood of working in a deprived area in the future among the studied medical students (n = 372).

Likelihood of working in a deprived area in the future (n = 323)	n			%
I will definitely not work in a deprived area	100			26.9%
I am unlikely to work in a deprived area	181			48.7%
I am likely to work in a deprived area	38			10.2%
I will definitely work in a deprived area	4			1.1%
Reasons for the likelihood of working in a rural area in the future	1^st^ (n = 41)	2^nd^ (n = 40)	3^rd^ (n = 39)	Relative score
To serve humanity	2.7%	1.9%	1.3%	2.2%
The feeling of connection & appreciation from the community	1.6%	1.9%	1.3%	1.7%
More opportunities to gain clinical experience	1.3%	1.9%	1.3%	1.5%
More cooperation from the community	1.1%	1.3%	1.1%	1.2%
I come from a rural area and feel at home there	1.1%	1.6%	0.5%	1.2%
The cost of living in the city is very high, and I can live more comfortably in the deprived area	1.3%	1.1%	0.5%	1.1%
Work is more exciting/challenging	0.5%	0.5%	1.6%	0.7%
Deprived area incentives	0.8%	0.3%	0.5%	0.6%
Deprived service increases my chances of going for further studies	0.5%	0.3%	1.6%	0.6%
A deprived area community has supported me financially during my training	0.0%	0.0%	0.5%	0.1%
Reasons for the unlikelihood of working in a rural area in the future	1^st^ (n = 304)	2^nd^ (n = 303)	3^rd^ (n = 302)	Relative score
Poor quality of clinic facilities	16.4%	13.7%	9.1%	14.3%
Lack of training opportunities	18.3%	9.4%	6.7%	13.4%
Limited career progression opportunities	9.7%	10.5%	12.4%	10.4%
Insufficient financial incentives	9.9%	7.5%	7.3%	8.7%
Lack of social amenities	6.7%	4.6%	7.3%	6.1%
Poor quality of housing	5.9%	5.1%	5.9%	5.6%
Poor quality of education for children	3.8%	6.5%	7.8%	5.4%
Insufficient professional support and mentorship	1.9%	8.3%	6.2%	4.8%
Little access to centralized employment-related support	2.2%	3.0%	3.0%	2.6%
Cut off from information sources (e.g., scholarships and promotion opportunities)	0.5%	4.0%	4.6%	2.4%
Little employment opportunities for spouse/partner	1.6%	2.7%	3.2%	2.2%
Lack of travel opportunities	0.8%	1.3%	1.3%	1.1%
Difficult to return to school for further education	0.5%	1.1%	1.6%	0.9%
Language barrier	0.3%	1.6%	1.1%	0.9%
Mentors and teachers advise against it	0.3%	0.8%	0.5%	0.5%
Other	3.0%	1.3%	3.2%	2.5%

Among students who expressed willingness to work in a deprived area (n = 41), the main motivating factors were altruistic and experiential. These included serving humanity (9, 22.0%), feeling a sense of connection and appreciation from the community (7, 17.1%), and opportunities to gain clinical experience (6, 14.6%). Other motivating factors included cooperation from the community (5, 12.2%), rural background (5, 12.2%), lower cost of living (4, 9.8%), and the perception that work is more exciting or challenging (3, 7.3%). Deprived area incentives and increased chances for further studies were less frequently cited, each by two (4.9%) students.

In contrast, students who were unlikely to work in a deprived area (n = 304) cited several barriers. The most influential deterrents were poor quality of clinic facilities (44, 14.3%), lack of training opportunities (41, 13.4%), and limited career progression opportunities (32, 10.4%). Financial concerns were also important, with 27 (8.7%) citing insufficient financial incentives. Additional barriers included lack of social amenities (19, 6.1%), poor housing (17, 5.6%), and inadequate education for children (16, 5.4%). Less frequently cited barriers included insufficient professional support and mentorship (15, 4.8%), limited access to employment-related support (8, 2.6%), restricted travel opportunities (3, 1.1%), language barriers (3, 0.9%), and other reasons (8, 2.5%).

As shown in Table 8, the most commonly cited incentive was a base salary plus 30% or greater, reported by 223 (59.9%) students. Other highly important conditions included a supportive workplace and management, cited by 172 (46.2%), and advanced infrastructure, equipment, and medical supplies, reported by 171 (46.0%).

**Table 8:** Incentives or conditions that medical students consider necessary to accept a posting in a deprived area (n = 372).

Incentives or conditions	n	%
Base salary plus 30% or greater	223	59.9%
Supportive workplace and management	172	46.2%
Advanced infrastructure, equipment, and supplies (e.g., reliable electricity, ultrasound, and constant drug supply)	171	46.0%
Allowance for children's education	127	34.1%
Free basic housing provided (shared with other health staff) or better	96	25.8%
None: no necessary benefits for me to work in a rural area	45	12.1%
Other	15	4.0%

Additional considerations included allowances for children’s education, indicated by 127 (34.1%) students, and free basic housing (shared with other health staff) or better, reported by 96 (25.8%). A small proportion of students (45, 12.1%) indicated that no incentives were necessary for them to work in a rural area, while other unspecified factors were reported by 15 (4.0%) students.

The ranking of the importance of career-influencing factors among medical students was done based on scores given to each factor, with a higher score indicating more importance (Table 9). The results show that salary is the most influential career factor for medical students, receiving the highest mean score (5.0 ± 2.0). This is followed by infrastructure, equipment, and supplies (mean = 4.6 ± 2.0) and children’s education (mean = 4.3 ± 1.7), showing that students place substantial value on adequate resources and family-related considerations. Management style and housing received moderate mean scores (4.2 ± 1.8 and 4.1 ± 1.6, respectively), while transportation (3.5 ± 1.9) and minimum years of work before study leave (3.2 ± 2.2) were rated lowest, indicating that these factors are relatively less influential.

**Table 9:** Ranking of career-influencing factors among the medical students (n = 372). Data are presented as median (IQR) and mean ± SD.

Career-influencing factors in order of their importance	n	Median (IQR)	Mean ± SD
Salary	282	6 (5)	5.0±2.0
Infrastructure, equipment, and supplies	266	5 (4)	4.6±2.0
Children’s education	264	5 (4)	4.3±1.7
Management style	268	4 (4)	4.2±1.8
Housing	262	4 (3)	4.1±1.6
Transportation	266	3 (3)	3.5±1.9
Minimum years of work before study leave	274	3 (3)	3.2±2.2

In the univariate logistic regression analysis, several factors were significantly associated with the willingness to work in rural areas (Table 10). Current year in the university demonstrated a clear pattern, whereby students in the 3rd, 4th, and 5th years were significantly less willing to work in rural areas compared with 1st-year students. The odds of willingness were reduced for 3rd-year students (OR = 0.25, 95% CI: 0.08-0.82, p = 0.022), 4th-year students (OR = 0.22, 95% CI: 0.05-0.87, p = 0.031), and 5th-year students (OR = 0.22, 95% CI: 0.08-0.62, p = 0.004). Age was also significant. Students aged 25-34 years were more likely to express willingness to work in rural areas compared with those aged 18-24 years (OR = 2.91, 95% CI: 1.20-7.09, p = 0.018). In addition, secondary school type showed a significant association, with public school graduates having higher odds of willingness compared to private school graduates (OR = 2.52, 95% CI: 1.27-4.98, p = 0.008). Students who had ever lived in a rural area were also more likely to express willingness to work in a rural setting (OR = 2.77, 95% CI: 1.37-5.60, p = 0.005).

**Table 10:** Logistic regression analysis for factors associated with the willingness to work in rural areas. OR: odds ratio; CI: confidence interval; Ref: reference category; * statistical significance at p-value < 0.05.

Item	Univariate analysis			Multivariable analysis		
	Unadjusted OR	95% CI of unadjusted OR	P-value	Adjusted OR	95% CI of adjusted OR	P-value
Current year in the university						
1^st^ year	Ref			Ref		
2^nd^ year	0.32	0.10 to 1.05	0.061	0.32	0.09 to 1.11	0.074
3^rd^ year	0.25	0.08 to 0.82	0.022*	0.22	0.06 to 0.77	0.018*
4^th^ year	0.22	0.05 to 0.87	0.031*	0.24	0.05 to 1.07	0.061
5^th^ year	0.22	0.08 to 0.62	0.004*	0.15	0.05 to 0.47	0.001*
Internship	0.42	0.16 to 1.13	0.085	0.26	0.07 to 0.94	0.04*
Gender						
Male	Ref			Ref		
Female	1.3	0.68 to 2.50	0.424	1.7	0.76 to 3.78	0.196
Age						
18-24	Ref			Ref		
25-34	2.91	1.20 to 7.09	0.018*	4.14	1.19 to 14.43	0.026*
Birth region						
Central Region	Ref			Ref		
Eastern Region	2.16	0.66 to 7.04	0.2	1.56	0.43 to 5.68	0.5
Western Region	1.24	0.34 to 4.47	0.742	0.78	0.16 to 3.85	0.76
Northern Region	3.52	0.62 to 19.98	0.156	1.03	0.14 to 7.8	0.976
Southern Region	0.41	0.05 to 3.21	0.399	0.18	0.02 to 1.64	0.128
Secondary school						
Private	Ref			Ref		
Public	2.52	1.27 to 4.98	0.008*	1.88	0.89 to 3.98	0.098
Currently married						
No	Ref			Ref		
Yes	2.83	0.53 to 15.09	0.223	2.64	0.43 to 16.14	0.293
Ever lived in a rural area						
No	Ref			Ref		
Yes	2.77	1.37 to 5.60	0.005*	3.11	1.37 to 7.07	0.007*

After adjusting for all included covariates in the multivariable logistic regression model, several associations remained statistically significant. Current year in the university continued to show a significant effect, with 3rd-year students (adjusted OR = 0.22, 95% CI: 0.06-0.77, p = 0.018) and 5th-year students (adjusted OR = 0.15, 95% CI: 0.05-0.47, p = 0.001) being less willing to work in rural areas compared with 1st-year students. Interns also showed significantly lower willingness (adjusted OR = 0.26, 95% CI: 0.07-0.94, p = 0.040). Age remained a strong predictor, with students aged 25-34 years demonstrating significantly higher willingness compared with those aged 18-24 years (adjusted OR = 4.14, 95% CI: 1.19-14.43, p = 0.026). Ever having lived in a rural area also remained independently associated with higher willingness (adjusted OR = 3.11, 95% CI: 1.37-7.07, p = 0.007). Meanwhile, the secondary school type was no longer statistically significant after adjustment (adjusted OR = 1.88, 95% CI: 0.89-3.98, p = 0.098), suggesting that its univariate association was confounded by other variables in the model.

## Discussion

Major findings

The main finding of this study is the very low intention among medical students at King Saud University (KSU) to work in rural practice, with only 42 (11.3%) expressing a positive intention (38 (10.2%) “likely” and four (1.1%) “definitely will”). This lack of positive intention toward rural practice is further reflected in the strong preference for urban practice, as 302 (81.2%) of participants reported intending to work in urban areas after completing their studies, whereas only three (0.8%) intended to practice in rural areas.

The factors that emerged most strongly as deterrents to rural practice were predominantly systemic and professional in nature, including suboptimal quality of clinical infrastructure (44, 14.3%), lack of training opportunities (41, 13.4%), and limited opportunities for career advancement (32, 10.4%). From a financial perspective, insufficient financial incentives (27, 8.7%) were also identified as an important deterrent. Conversely, the most highly rated incentives for accepting a rural posting were a basic salary increment of 30% or more (223, 59.9%), a supportive workplace and management environment (172, 46.2%), and improved infrastructure, equipment, and medical supplies (171, 46.0%).

In the overall ranking of factors influencing career decisions, salary (mean rank: 5.04) and infrastructure, equipment, and supplies (mean rank: 4.59) were identified as the most influential determinants. Multivariable logistic regression analysis revealed three significant predictors of willingness to practice in rural areas: academic year, age, and prior rural residential experience. Students in more advanced academic stages (fifth year and internship) were significantly less willing to work in rural areas compared with first-year students, whereas older students (aged 25-34 years) and those with prior rural residential experience were significantly more willing to consider rural practice.

Interpretation of major findings

The unwillingness of KSU medical students to work in rural areas can be conceptualized within the context of perceived disparities in professional and personal quality of life between urban and rural settings in Saudi Arabia. Given that KSU is located in Riyadh, the capital city of the Kingdom, it is not surprising that students demonstrate a strong preference for urban environments, particularly as 362 (97.3%) of the study population currently reside in urban areas.

Deterrents such as inadequate facilities, limited training opportunities, and restricted career development appear to be closely linked to students’ professional ambitions and desire to pursue high-standard medical careers. The presence of major incentives, such as compensation in the form of a 30% or greater salary and improved infrastructure, further reinforces the perception of disadvantage and the need for a more compelling compensation package to attract them to rural locations. Students in their later years seem less favorable about rural locations, suggesting a certain shift with greater awareness about their field and familiarity with the realities of the landscape, tending toward urban settings with the best resources. The positive association with their prior residence attests to the importance of awareness and familiarity, suggesting potential experience as capable of countering any disadvantages posed by rural location.

Significance

These findings have important implications for healthcare workforce planning in Saudi Arabia, particularly in the context of Saudi Vision 2030. The focus of talent in the urban sector further exacerbates the challenge of misdistribution of the healthcare force [9,10], which remains an ever-unfolding challenge globally and requires a comprehensive approach. This study provides a key guiding light on the exact professional and infrastructure deficits that discourage physicians-to-be and can play a pivotal role in guiding effective policy responses. Considering these points will not only help in mitigation and remedial measures but will also ensure that all people across the geographical boundaries have equal access to quality healthcare.

Comparison with the literature

This study found that inadequate infrastructure and limited training opportunities were among the strongest deterrents to rural practice. These findings are consistent with existing international and regional literature on the recruitment and retention of healthcare professionals in rural settings. In the global context, professional factors such as access to career development opportunities, availability of adequate infrastructure, and exposure to high-quality training have been shown to play a more influential role in discouraging rural practice than personal or demographic factors alone [11,12].

The association between prior rural exposure and increased willingness to practice in rural areas has been well documented across diverse healthcare systems. Multiple international studies have demonstrated that students with rural backgrounds or those who undergo rural training experiences during their undergraduate education are more likely to choose rural practice after graduation [13,14]. The current study confirms that this association holds true within the Saudi context, suggesting that the underlying relationship between exposure and career choice operates similarly despite differences in healthcare systems and cultural settings.

Furthermore, the reduced willingness to practice in rural areas observed among students in more advanced academic years supports findings from other countries with centralized, urban-based medical education models. Studies have shown that prolonged exposure to tertiary care hospitals in major urban centers may reinforce preferences for metropolitan practice and diminish interest in rural careers over time [15,17]. This trend highlights the potential unintended consequences of predominantly urban clinical training environments and underscores the importance of early and sustained rural exposure during medical education.

Taken together, these findings suggest that while the determinants of rural practice intention among medical students in Saudi Arabia are broadly consistent with global patterns, the magnitude of perceived disparities between urban and rural practice environments, particularly with respect to infrastructure and training quality, may be especially pronounced. Addressing these gaps through targeted educational reforms and policy interventions is therefore essential to improving rural workforce distribution.

Clinical implications

The findings of this study highlight several important implications for policymakers and medical educators aiming to address the rural healthcare workforce gap in Saudi Arabia. First, tailored financial and non-financial incentive packages are essential. These should extend beyond salary increments to include improved clinical infrastructure, availability of essential equipment and supplies, and structured opportunities for career development during rural service.

Second, the integration of mandatory, high-quality rural exposure within the medical curriculum is critical. Early exposure during pre-clinical and early clinical years, designed to emphasize positive learning experiences and professional growth, may improve willingness to practice in rural areas [14,18].

Third, support mechanisms addressing family-related concerns, including housing quality and children’s education, should be incorporated into rural recruitment strategies to make rural practice a viable option for physicians with families.

Finally, decentralization of postgraduate medical training is necessary to prevent rural practice from being perceived as a career dead-end. Establishing accredited residency and fellowship programs in rural hospitals could enhance professional satisfaction and long-term retention of physicians in underserved areas.

Strengths, limitations, and future directions

This study has several strengths. It addresses an important public health issue related to rural health workforce distribution in Saudi Arabia and targets medical students and interns at a critical career decision point. The inclusion of participants across different academic years and the use of multivariable analysis allowed for adjustment of potential confounders and strengthened the internal validity of the findings.

Several limitations are noted in this study. Firstly, the cross-sectional design and the inclusion of participants from a single, highly urbanized institution may limit the generalizability of the findings to medical students in other regions of Saudi Arabia, particularly those studying in less urbanized settings. Secondly, the study relies on self-reported intentions and preferences, which may not accurately predict actual career choices following graduation.

In addition, the use of a self-administered questionnaire and voluntary participation may introduce measurement and selection bias. The relatively small proportion of students with rural living or work experience may have reduced the statistical power to detect associations within these subgroups.

Future research should include medical students from multiple universities across different regions of the Kingdom to provide a more comprehensive national perspective. Longitudinal studies following graduates into their professional careers would help assess whether stated intentions translate into actual rural practice. Furthermore, qualitative research methods, such as in-depth interviews or focus group discussions with medical students and rural practitioners, may offer deeper insights into the complex factors influencing decisions to work in rural areas.

## Conclusions

Medical students at King Saud University show low willingness to practice in rural areas. Addressing systemic barriers, improving rural infrastructure, enhancing financial incentives, and incorporating structured rural training into medical education may help support more equitable healthcare distribution in Saudi Arabia.
